# The stem/progenitor landscape is reshaped in a mouse model of essential thrombocythemia and causes excess megakaryocyte production

**DOI:** 10.1126/sciadv.abd3139

**Published:** 2020-11-25

**Authors:** Daniel Prins, Hyun Jung Park, Sam Watcham, Juan Li, Michele Vacca, Hugo P. Bastos, Alexander Gerbaulet, Antonio Vidal-Puig, Berthold Göttgens, Anthony R. Green

**Affiliations:** 1 Wellcome-MRC Cambridge Stem Cell Institute, University of Cambridge, Cambridge, UK.; 2 Department of Haematology, University of Cambridge, Cambridge, UK.; 3 Wellcome Trust–Medical Research Council Institute of Metabolic Science-Metabolic Research Laboratories, Addenbrooke’s Hospital, Cambridge, UK.; 4 Institute for Immunology, Faculty of Medicine Carl Gustav Carus, Technische Universität Dresden, Dresden, Germany.; 5 Wellcome Trust Sanger Institute, Hinxton, UK.

## Abstract

Frameshift mutations in *CALR* (calreticulin) are associated with essential thrombocythemia (ET), but the stages at and mechanisms by which mutant CALR drives transformation remain incompletely defined. Here, we use single-cell approaches to examine the hematopoietic stem/progenitor cell landscape in a mouse model of mutant CALR-driven ET. We identify a trajectory linking hematopoietic stem cells (HSCs) with megakaryocytes and prospectively identify a previously unknown intermediate population that is overrepresented in the disease state. We also show that mutant CALR drives transformation primarily from the earliest stem cell compartment, with some contribution from megakaryocyte progenitors. Last, relative to wild-type HSCs, mutant CALR HSCs show increases in JAK-STAT signaling, the unfolded protein response, cell cycle, and a previously undescribed up-regulation of cholesterol biosynthesis. Overall, we have identified a novel megakaryocyte-biased cell population that is increased in a mouse model of ET and described transcriptomic changes linking *CALR* mutations to increased HSC proliferation and megakaryopoiesis.

## Teaser

Mutant CALR drives ET at multiple stages of hematopoiesis.

## INTRODUCTION

The myeloproliferative neoplasms are a family of clonal blood disorders characterized by overproduction of platelets [essential thrombocythemia (ET)], overproduction of red blood cells [polycythemia vera (PV)], or bone marrow fibrosis [myelofibrosis (MF)]. The genetic bases for these diseases have largely been described: Mutations in *JAK2* are found in 99% of PV and 50 to 60% of ET and MF cases, while frameshift mutations in *CALR* are responsible for 25 to 40% of cases of ET and MF (*1*–*3*). Frameshift mutants of calreticulin (CALR) have a novel C terminus that acts as a rogue ligand for the thrombopoietin receptor, *MPL*, and activates Janus kinase–signal transducer and activator of transcription (JAK-STAT) signaling (*4*, *5*). We recently described the generation of a mouse model of mutant *CALR*-driven ET that faithfully recapitulates the key phenotypes of the human disease, namely, increased numbers of cells throughout the megakaryocytic (MK) lineage, particularly platelets (*6*).

Hematopoiesis is classically modeled as a stepwise process beginning with a multipotent hematopoietic stem cell (HSC), which is functionally defined by its capability to reconstitute multilineage hematopoiesis when transplanted into a myeloablated recipient (*7*). This HSC then transits through a series of intermediate stages with increasing lineage restriction to terminally differentiated blood cells (*8*, *9*). However, newly popularized single-cell technologies such as single-cell RNA sequencing (scRNAseq) have reshaped our understanding of hematopoiesis and suggest that cells travel through a continuum of differentiation rather than a series of rigidly defined stages (*10*, *11*). In a recent demonstration of the power of scRNAseq to untangle complex differentiation processes, it was used to interrogate the transcriptomes of hematopoietic stem and progenitor cells (HSPCs) to identify novel intermediate populations within erythropoiesis, which could then be isolated and characterized via fluorescence-activated cell sorting (FACS) strategies (*12*).

While HSCs are traditionally defined to be capable of reconstituting all blood lineages in transplantation experiments, there is an increasing body of evidence that some cells within the immunophenotypic HSC compartment already exhibit some lineage bias or restriction (*13*–*15*). Studies in mice have shown that MK and erythroid lineages may branch off before other myeloid and lymphoid lineages (*16*–*18*), and lineage tracing studies have shown the MK lineage to be the earliest generated from HSCs (*19*–*23*). A transposon-based lineage tracing strategy showed some tags to be shared between long-term HSCs (LT-HSCs) and megakaryocyte progenitors (MkPs) but not multipotent progenitors (MPPs), indicative of a direct pathway linking HSCs and MK bypassing MPP (*19*). We therefore asked whether our mouse model of mutant *CALR*-driven ET could allow us to interrogate the differences in the hematopoietic landscapes between wild-type (WT) and disease model mice, with a particular focus on MK trajectories.

## RESULTS

### scRNAseq identifies a novel cell population overproduced in mutant CALR mice

We generated scRNAseq data from FACS-sorted HSPCs [Lin^−^ Sca1^+^ cKit^+^ (LSK) and Lin^−^ Sca1^−^ cKit^+^ (LK) populations] from a pair of WT and *CALR DEL* (knock-in of del52 allele) homozygous (HOM) littermate mice. After quality control, we retained 11,098 WT (5959 LSK and 5139 LK) and 15,547 HOM (7732 LSK and 7815 LK) cells for downstream analysis. We began by defining highly variable genes, which we used to perform principal component analysis (PCA) and generate a *k* = 7 nearest-neighbor graph. Cells were then assigned to clusters by mapping onto a previously published dataset of 44,082 LK cells (*24*), with manual annotation of clusters (fig. S1A). Cells from all major blood lineages can be seen and separate into distinct trajectories. To determine which cells were over- or underrepresented in the *CALR* DEL HOM mouse, we compared relative numbers of cells from each genotype. The most notable changes in relative cell abundance were increased numbers of cells in the HSC and MK clusters (fig. S1B), consistent with the increased platelet phenotype of our ET mouse model (*6*). We repeated the analysis on a second pair of WT and *CALR* DEL HOM littermate mice, in this case retaining 3451 WT (972 LSK and 2479 LK) and 12,372 HOM (4548 LSK and 7824 LK) cells for downstream analysis after quality control, and again observed an increase in cells in the HSC and MK clusters (fig. S1C).

**Fig. 1:**
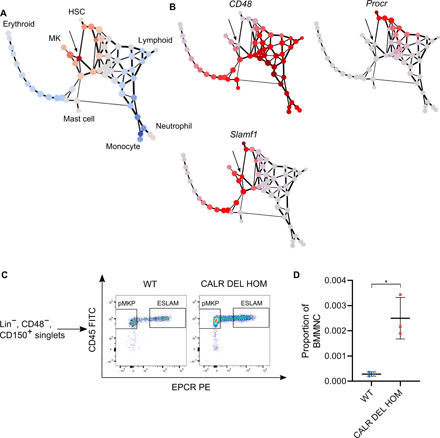
An MK trajectory is increased in *CALR* DEL HOM mice. (**A**) PAGA of scRNAseq data from WT and *CALR* DEL HOM mice. Red nodes represent those present at increased abundance in *CALR* DEL HOM mice, while blue nodes represent those at reduced abundance. The most highly enriched node is noted with an arrow. (**B**) RNA expression of the flow cytometry markers CD48, EPCR (*Procr*), and CD150 (*Slamf1*) plotted on PAGA graphs from (A). Cells within our node of interest (marked with an arrow) are CD48^−^, EPCR^−^, and CD150^+^. (**C**) Representative plots of SLAM cells from WT and *CALR* DEL HOM mice. *CALR* DEL HOM mice show higher numbers of both ESLAMs (Lin^−^ CD48^−^ CD150^+^ CD45^+^ EPCR^+^) and pMKPs (Lin^−^ CD48^−^ CD150^+^ CD45^+^ EPCR^−^). FITC, fluorescein isothiocyanate; PE, phycoerythrin. (**D**) Quantification of bone marrow frequency of pMKPs in WT and *CALR* DEL HOM mice. The frequency of pMKPs within live bone marrow mononuclear cells (BMMNCs) is significantly increased in *CALR* DEL HOM mice (WT, *n* = 3, 0.00029 ± 0.00008; HOM, *n* = 3, 0.0025 ± 0.0008; *P = 0.042).

To better understand the subgroups of cells within stem/progenitor cells, we chose to use partition-based graph abstraction (PAGA) (*25*) to visualize our data. This method generates a graph in which each node represents a group of closely related cells and edge weights correspond to the strength of connection between two nodes. We again compared relative abundances between WT and *CALR* DEL HOM mice and colored the nodes so red nodes are enriched in *CALR* mice, while blue nodes are underrepresented. We observed that the fine cluster that was most overrepresented in *CALR* DEL HOM mice (marked with an arrow) fell between the HSC and MK clusters in both repeats (Fig. 1A and fig. S1D). We plotted the expression of the MK markers *Cd9*, *Itga2b* (CD41), *Mpl*, *Pf4*, and *VWF* in our PAGA and hypothesized two MK trajectories, as indicated by the green and blue arrows (fig. S1E). As the fine cluster most overrepresented in *CALR* DEL HOM mice would be an intermediate on one of these trajectories (green arrow), we further hypothesized that these cells would be of particular relevance in the disease setting of mutant *CALR*-driven ET and thus aimed to further study them.

We examined the expression of a series of genes typically used to FACS isolate different hematopoietic populations and found this fine cluster to be CD48^−^, EPCR^−^ (*Procr*), and CD150^+^ (*Slamf1*) (Fig. 1B). We designed an immunophenotypic scheme to identify and isolate cells from this fine cluster, defining them to be Lin^−^, CD150^+^, CD48^−^, EPCR^−^, and CD45^+^. On the basis of our subsequent characterization of these cells, we eventually termed them “proliferative MkPs” or pMKPs. Consistent with our transcriptomic data, when comparing WT mice to CALR mutant mice, we found an increase in the frequency of pMKPs in *CALR* DEL HOM mice as assayed by flow cytometry (Fig. 1, C and D). We also found that pMKPs were expanded in *CALR* DEL HET mice, albeit to a lesser extent than observed in *CALR* DEL HOM mice (fig. S1F).

### pMKPs are proliferative and MK-biased

**Fig. 2:**
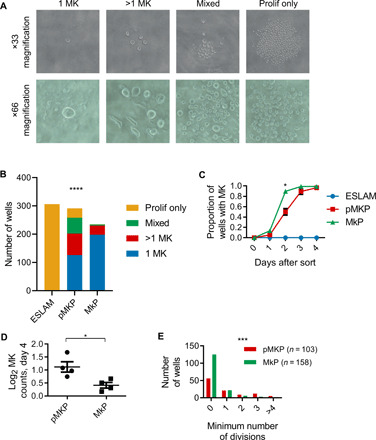
pMKPs are MK-biased and proliferative in vitro. (**A**) Representative pictures of in vitro culture output of single ESLAMs, pMKPs, and MkPs into four categories: 1 MK, >1 MK, mixed, or proliferation only. (**B**) Classification of in vitro culture output of single ESLAMs, pMKPs, and MkPs at day 4 after FACS isolation. ESLAMs almost exclusively proliferated without producing megakaryocytes, while MkPs almost exclusively produced MKs, usually producing only a single MK. pMKPs showed a strong MK bias but were more likely to proliferate than were MkPs. ESLAMs, *n* = 306 wells from five experiments; pMKPs, *n* = 291 wells from six experiments; MkPs, *n* = 235 wells from five experiments. Chi-square test, ****P < 0.0001. (**C**) Timing of megakaryopoiesis in ESLAMs, pMKPs, and MkPs. Individual cells were observed for 4 days after sort, and the first date on which cell(s) showed signs of megakaryopoiesis was noted. MkPs were faster to begin megakaryopoiesis than were pMKPs (at day 2, MkPs: 89.5 ± 0.7%; pMKPs: 50 ± 6%; *P = 0.02). ESLAMs, *n* = 5; pMKPs, *n* = 6; MkPs, *n* = 5. (**D**) Log_2_-transformed cell counts of megakaryocytes from pMKPs and MkPs after 4 days of culture. Each point represents the average value from one of four separate experiments. Average of four experiments: pMKP, 1.12; MkP, 0.412, *P = 0.0295. (**E**) Histogram of the minimum number of cell divisions for 103 pMKPs and 158 MkPs that produced only megakaryocytes after 4 days of culture across four experiments. Chi-square test, ***P = 0.0001.

To characterize pMKPs, we FACS-sorted single ESLAM (EPCR^+^ SLAM) HSCs (Lin^−^ CD45^+^ CD48^−^ CD150^+^ EPCR^+^) (*26*), pMKPs (Lin^−^ CD45^+^ CD48^−^ CD150^+^ EPCR^−^), and MkPs (Lin^−^ Sca1^−^ cKit^+^ CD41^+^ CD150^+^) (*27*) (fig. S2A) from WT mice into individual wells of a 96-well plate and observed them every day for 4 days. We analyzed our sort data and observed that in pMKPs, markers traditionally used to define MkPs were Sca1^−/lo/mid^, cKit^+^, and CD41^mid/+^ (fig. S2B). pMKPs were additionally CD9^+^ and MPL^+^ (fig. S2C). On each day, we classified each well with surviving cell(s) into one of four categories, using cell size as a proxy for megakaryopoiesis (*28*–*30*): (i) exactly one large cell, presumed to be a megakaryocyte; (ii) multiple large cells; (iii) mixed expansion, with both large and small cells; and (iv) expansion with only small cells (Fig. 2A). To verify that larger cells represented MK cells, using cells from day 4 ESLAM, pMKP, and MkP colonies, we quantified average CD41 intensity via immunofluorescence and classified cells as small or large via bright-field microscopy, using a small/large dichotomy assessed via bright-field microscopy to match the classification scheme used in Fig. 2A. Here, we confirmed that large cells have significantly higher CD41 staining, supporting their identification as MK (fig. S2D). In some cases, particularly large cells within mixed colonies showed very high CD41 staining and membrane extensions that resembled proplatelets (representative picture is shown in fig. S2E). Furthermore, we sorted pMKPs from VWF (von Willebrand factor)–green fluorescent protein–positive (GFP^+^) mice and found that large cells had a very bright VWF-GFP signal, supporting their identification as MK. Smaller cells in these clones had a much dimmer VWF-GFP signal, suggesting that they likely represent more immature cells that have not progressed as far through megakaryopoiesis (fig. S2F).

The vast majority of ESLAMs showed expansion with only small cells at day 4, consistent with being highly primitive HSCs with considerable proliferative potential, but not yet producing megakaryocytes. Similarly, as predicted for MkPs, more than 95% of wells showed exclusively production of MKs at day 4, with the majority producing only one MK. This lack of in vitro proliferation for single MkPs is consistent with previously published results, where 75% of MkPs did not divide and none produced more than 10 MKs (*31*). pMKPs exhibited an intermediate phenotype: While approximately 90% of wells showed production of some MKs, they were much more likely to produce multiple MK than were MkPs. In particular, pMKPs frequently proliferated into mixed colonies with both large and small cells, a behavior that was rarely seen for either ESLAMs or MkPs (Fig. 2B). Kinetic analysis showed that MkPs were faster to begin megakaryopoiesis than were pMKPs (Fig. 2C), and when considering only wells that produced only MKs, pMKPs produced more MKs than did MkPs (Fig. 2, D and E). pMKPs maintained their MK bias even when incubated under pro-erythroid or pro-myeloid conditions (fig. S3A). Culturing cells with thrombopoietin (THPO) increased the proportion of pMKPs that formed colonies with multiple MKs while reducing the number of mixed colonies (fig. S3B). To verify that our observed MK bias is not simply due to culture conditions supporting only megakaryopoiesis, we cultured ESLAMs under the same conditions for 10 days followed by flow cytometric analysis and observed multilineage differentiation (fig. S3C).

To examine the extent of overlap between our pMKPs and traditionally defined MkPs, we stained bone marrow with a panel incorporating all necessary markers and index sorted single pMKPs and MkPs. On the basis of index sort values, 97% of MkPs were CD45^+^, 50% were EPCR^−^, and only 2% were CD48^−^; when taken together, fewer than 1% of immunophenotypic MkPs also fell within the pMKP gate (fig. S3D); thus, pMKPs and MkPs can be FACS-separated on the basis of CD48^−^ and EPCR^−^. In contrast, we found that an average of 51% of pMKPs were also immunophenotypically MkPs (CD41^+^ Sca1^−^ cKit^+^) (fig. S3E). As we observed a partial overlap between pMKPs and MkPs, we used our index sort data to assign each pMKP an overlap score based on the levels of CD41, Sca1, and cKit: 1/3 if only one marker overlapped, 2/3 if two overlapped, and 3/3 for pMKPs that also fall within the MkP immunophenotypic gate. No pMKPs had an overlap score of 0/3. We used the same classification scheme as in Fig. 2B and found that lower overlap scores correlated to a more proliferative, less MK-restricted phenotype: The pMKPs that are least similar to MkPs are the most proliferative and the least restricted to the MK lineage, although they still display a strong preference for MK production (fig. S3F). pMKPs with the lowest overlap score took the longest to enter megakaryopoiesis (fig. S3G). Together, our data indicate that pMKPs represent a group of cells with an MK bias and an increased proliferative potential as compared to traditionally defined MkPs.

### pMKPs show limited and short-term contribution to platelets in transplants

**Fig. 3:**
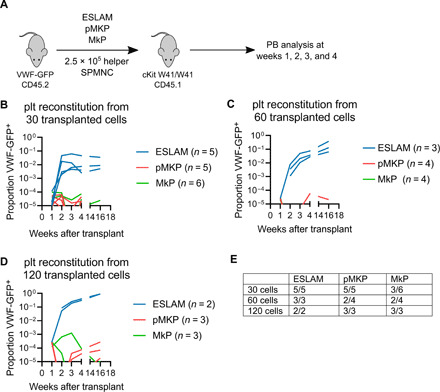
pMKPs have limited platelet potential in transplantations. (**A**) Schematic of VWF-GFP^+^ transplantation strategy. ESLAMs, pMKPs, and MkPs were sorted from VWF-GFP^+^, CD45.2 donor mice and transplanted into sublethally irradiated cKit W41/W41 CD45.1 recipients. PB, peripheral blood. (**B**) Platelet reconstitution from 30 donor cells. (**C**) Platelet reconstitution from 60 donor cells. (**D**) Platelet reconstitution from 120 donor cells. (**E**) Table summarizing numbers of mice with successful platelet production from ESLAMs, pMKPs, and MkPs. Here, transplanted cells were defined to have produced platelets if platelets were observed at a level of at least 1 in 10^5^ at one or more time points within the first 4 weeks after transplantation.

We next determined whether pMKPs were capable of producing platelets in vivo. We made use of CD45.2 VWF-GFP donor mice and cKit W41/W41 CD45.1 recipient mice, which allowed us to track platelets (via VWF-GFP) and nucleated cells (by CD45.1/CD45.2 staining) (Fig. 3A). We FACS-sorted ESLAMs, pMKPs, and MkPs from VWF-GFP donor mice and transplanted 30, 60, or 120 cells per recipient into sublethally irradiated W41 mice along with 250,000 spleen MNCs (mononuclear cells) (SPMNCs) as helper cells and assayed peripheral blood chimerism every week for 4 weeks and at 16 weeks. We did not sort on VWF-GFP^+^ at this stage, but flow cytometry analysis showed that ESLAMs, pMKPs, and MkPs were all highly enriched for VWF-GFP expression when compared to total bone marrow (fig. S4A). We also transplanted one mouse per cohort with 250,000 SPMNCs alone to serve as a negative control to help with gating to avoid false positives. Representative gating strategies are shown in fig. S4 (B and C). As expected, ESLAMs were able to generate relatively high levels of platelets at all three cell doses, starting with a very low level at week 1 and increasing over the course of 4 weeks and continuing up to 16 weeks (although one recipient of 30 ESLAMs was lost to follow-up before the 16-week time point). pMKPs and MkPs were only able to reconstitute platelets at a very low level (1/10^5^ to 1/10^4^), even at the highest cell dose (Fig. 3, B to D and summarized in E). Low levels of donor-derived platelets were detected in 10 of 12 pMKP recipients and 8 of 13 MkP recipients within the first 4 weeks; extended observation up to 16 weeks showed that few recipients continued to produce VWF-GFP^+^ platelets, although all 3 pMKP recipients at the highest dose still showed VWF-GFP^+^ platelets. ESLAMs successfully produced CD11b^+^ myeloid cells in 10 of 10 recipients across varying cell doses, while pMKPs and MkPs only produced CD11b^+^ cells at a low level in 3 of 12 and 2 of 10 recipients, respectively (fig. S4, D to F and summarized in G). Therefore, we concluded that while pMKPs and MkPs have limited capabilities in a transplantation experiment, they both show an MK bias, in agreement with their in vitro behaviors. These low levels of reconstitution suggest that pMKPs and MkPs do not divide considerably in vivo, again similar to in vitro data.

### pMKPs are produced from HSCs in an MPP2-independent manner

**Fig. 4:**
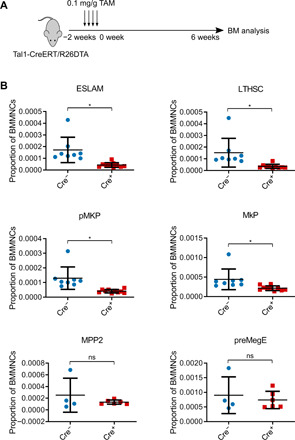
pMKPs originate from HSCs in a MPP2-independent manner. (**A**) Schematic of DTA (diphtheria toxin fragment A) HSC depletion model experiment. Tal1-CreERT/R26DTA mice were treated with four doses of tamoxifen at 0.1 mg/g to induce suicidal depletion of HSCs and then euthanized after 6 weeks for bone marrow (BM) analysis. (**B**) Frequencies of stem and progenitor cells with or without stem cell depletion. Cell populations that were significantly diminished by suicidal depletion of HSCs include ESLAMs (Cre^−^, 17.1 ± 10.8/10^5^ BMMNC; Cre^+^, 4.3 ± 2.0/10^5^ BMMNC; *P = 0.012), LTHSCs (LSK CD48^−^ CD150^+^) (Cre^−^, 15 ± 12/10^5^ BMMNC; Cre^+^, 3.6 ± 1.7/10^5^ BMMNC; *P = 0.031), pMKPs (Cre^−^, 13.0 ± 7.6/10^5^ BMMNC; Cre^+^, 4.1/10^5^ BMMNC; *P = 0.013), and MkPs (Cre^−^, 44.2 ± 26.4/10^5^ BMMNC; Cre^+^, 21.4 ± 6.1/10^5^ BMMNC; *P = 0.046); Cre^−^, *n* = 8 and Cre^+^, *n* = 10. MPP2 (Cre^−^, 25.1 ± 29.1/10^5^ BMMNC; Cre^+^, 13.3 ± 3.6/10^5^ BMMNC; *P* = 0.48) and preMegE (Cre^−^, 90.0 ± 62.9/10^5^ BMMNC; Cre^+^, 73.9 ± 29.6/10^5^ BMMNC; *P* = 0.66) populations were depleted to lesser extents that did not reach statistical significance; Cre^−^ *n* = 4 and Cre^+^ *n* = 6. ns, not significant.

Our single-cell transcriptomic analysis showed pMKPs to be an intermediate stage on an MK trajectory maintaining CD48 negativity (Fig. 1B and green arrow in fig. S1E), which suggests that they bypass the traditional MPP2 pathway (blue arrow in fig. S1E). We therefore asked whether we could show production of pMKPs from HSCs in an MPP2-independent manner by making use of a mouse model allowing inducible depletion of HSPCs. In this model, Tal1-Cre/ERT mice are crossed with R26^DTA^ mice, wherein treatment with tamoxifen leads to specific expression of diphtheria toxin in HSCs and primitive progenitors and hence suicidal depletion of these early populations (Fig. 4A) (*32*). Within 6 weeks after HSC depletion, very few LT-HSCs remain, but levels of MPPs, committed progenitors, and mature blood cells are only slightly lower than in control animals (*32*). We reasoned that if pMKPs arise directly from HSCs, they should be depleted to a similar extent as HSCs, while if they arise from an MPP pathway, they should be depleted to a similar extent as MPPs (i.e., to a lesser extent than HSCs).

We compared mice carrying either no Cre or Tal1-Cre/ERT after treatment with tamoxifen to induce specific depletion of HSCs. We observed a depletion of approximately 75% in the numbers of HSCs [whether using ESLAM markers or LT-HSC (LSK CD48^−^ CD150^+^) markers] and a 68% reduction in the numbers of pMKPs in HSC-depleted mice. By contrast, there was no significant reduction in MPP2 or preMegE populations, while MkPs were reduced by approximately 51% (Fig. 4B). Consistent with previously published results, we observed no statistically significant reduction in other multipotent populations, including MPP3 and MPP4 (*33*), and committed progenitor populations, including CFU-E (erythroid colony-forming units), pCFU-E, pGM (pre-granulocyte/macrophage), and GMP (granulocyte/monocyte progenitors) (fig. S5) (*27*). We noted that one Cre^−^ mouse was an outlier, with noticeably higher frequencies of almost all progenitor populations, and tested removing this outlier to ensure our conclusions were not unduly relying on this mouse. With the outlier removed, we calculated reductions of 68% in ESLAMs (*P* = 0.0001), 60% in pMKPs (*P* = 1.5 × 10^−5^), and an increase of 24% in MPP2 (*P* = 0.50). Our analysis is therefore robust to the removal of this outlier and demonstrates that the reduction in pMKP levels correlates more closely to that of ESLAMs than that of MPP2. Together, these data support a model in which pMKPs are produced from HSCs in an MPP2-independent manner and MkPs can be generated from pMKPs or via MPP2, accounting for their intermediate level of reduction.

### *CALR* DEL drives platelet bias and proliferation at multiple stages

**Fig. 5:**
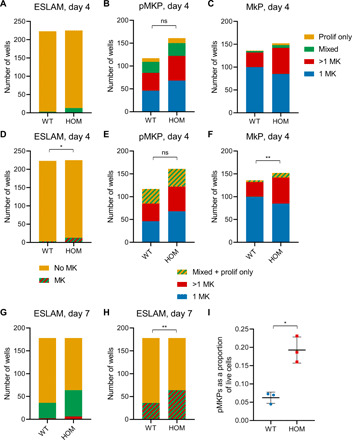
Mutant CALR drives an MK phenotype primarily from HSCs. (**A**) Classification of in vitro culture output of single ESLAMs from WT and *CALR* DEL HOM mice at day 4, using the classification scheme as in Fig. 2A. WT, *n* = 223; HOM, *n* = 225. (**B**) Classification of in vitro culture output of single pMKPs from WT and *CALR* DEL HOM mice at day 4; WT, *n* = 117; HOM, *n* = 161. Chi-square test *P* = 0.9201. (**C**) Classification of in vitro culture output of single MkPs from WT and *CALR* DEL HOM mice at day 4; WT, *n* = 136; HOM, *n* = 152. (**D**) Reclassification of data from (A) into two categories (MK or no MK) for a Fisher’s exact test, *P = 0.0191. (**E**) Reclassification of data from (B) into three categories (1 MK, >1 MK, and mixed + prolif only) for a chi-square test, *P* = 0.8183. (**F**) Reclassification of data from (C) into three categories (1 MK, >1 MK, and mixed + prolif only) for a chi-square test, **P = 0.0069. (**G**) Classification of in vitro culture output of single ESLAMs at day 7; WT, *n* = 136; HOM, *n* = 152. (**H**) Reclassification of data from (G) into two categories (MK or no MK) for a Fisher’s exact test, **P = 0.0014. (**I**) pMKPs as a proportion of live cells generated from in vitro culture of WT and *CALR* DEL HOM ESLAMs, assessed at day 3. WT, 0.062 ± 0.015; HOM, 0.193 ± 0.036, *P = 0.0135, *n* = 3 independent mice.

After characterizing the pMKP population in WT mice, we next asked whether there were qualitative differences between WT and *CALR* DEL HOM cells along the MK trajectory and not solely a quantitative difference. To do so, we sorted single ESLAMs, pMKPs, and MkPs from WT and *CALR* DEL HOM mice and monitored their in vitro behavior over 4 days. While very few WT ESLAMs showed any MKs within the first 4 days after sort, a higher proportion of *CALR* DEL HOM ESLAMs showed MKs within mixed colonies (Fig. 5A). *CALR* DEL HOM pMKPs showed similar proportions of wells in each category (Fig. 5B), while *CALR* DEL HOM MkPs were more likely to form multiple MKs and less likely to form a single MK (Fig. 5C). To assess the statistical significance of these differences, using a Fisher’s exact or chi-square test required consolidation of our data into fewer categories, as some categories contained values that were too low (for example, for day 4 ESLAMs, the categories “1 MK” and “>1 MK” were 0 in both WT and HOM). We thus consolidated ESLAM data into two categories—“no MK” and “MK” (Fig. 5D)—and pMKP and MkP data into three categories—1 MK, >1 MK, and “mixed + prolif only” (Fig. 5, E and F). This showed that *CALR* DEL HOM ESLAMs were significantly more likely to form MKs (Fig. 5D). *CALR* DEL HOM pMKPs showed no statistically significant difference, suggesting no change in their MK bias or proliferative behavior compared to WT pMKPs (Fig. 5E). *CALR* DEL HOM MkPs were significantly more proliferative than were WT MkPs (Fig. 5F). We also extended our observation of ESLAM clones to day 7 and observed an even stronger increase in the production of megakaryocytes from *CALR* DEL HOM ESLAMs, an increase noted both in wells producing mixed clones and in those producing MK-only clones (Fig. 5, G and H).

We also considered log_2_-transformed cell counts from those wells with exclusively megakaryocytes (i.e., 1 MK and >1 MK). In some cases, we observed the death of a cell or cells over our 4-day observation period; to account for cell death, we used the maximum number of cells observed over these 4 days. Mann-Whitney *U* tests showed no significant difference for pMKPs but a significant increase in MK production from *CALR* DEL HOM MkPs (fig. S6, A and B). Similarly, calculations of the minimum number of divisions required to produce the observed number of MKs found no difference for pMKPs but a significant shift to more divisions from *CALR* DEL HOM MkPs (fig. S6, C and D). We also cultured ESLAMs in vitro and assayed for the production of pMKPs, finding that *CALR* DEL HOM ESLAMs gave rise to significantly more pMKPs than did their WT counterparts (Fig. 5I). Together, we conclude that *CALR* DEL is acting at multiple stages of megakaryopoiesis, promoting an MK bias from the earliest HSC compartments and increased proliferation at both HSC and MkP levels. While pMKPs are increased in number in *CALR* DEL HOM mice, these cells do not show altered proliferation or MK bias in vitro.

### Transcriptomic analysis of CALR mutant cells shows dysregulated cholesterol biosynthesis

**Fig. 6:**
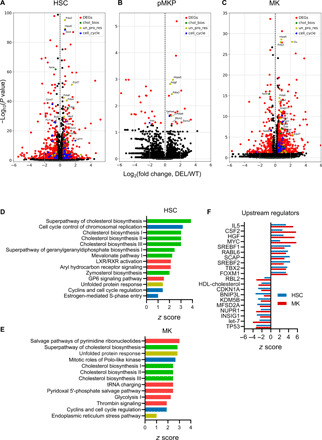
Mutant CALR affects STAT signaling, protein folding, cell cycle, and cholesterol biosynthesis. (**A** to **C**) Volcano plots showing DEGs between WT and *CALR* DEL HOM cluster 3 (HSC) (A), pMKP fine cluster (B), and cluster 11 (MK) (C). Genes within certain representative Gene Ontology (GO) terms are colored: regulation of cholesterol biosynthetic process (GO:0045540) (green), response to ER stress (GO:0034976) (gold), and regulation of mitotic cell cycle (GO:0007346) (blue). Other DEGs are colored in red. (**D** and **E**) Bar graphs showing *z* scores for up-regulated canonical pathways in cluster 3 (HSC) (C) and cluster 11 (MK) (D), filtered by *P* < 0.01 and *z* score of >1 or <−1. Bars are highlighted in green for cholesterol biosynthesis, gold for ER stress/unfolded protein response, or blue for cell cycle. (**F**) Upstream regulator analysis. Hits were filtered by *P* < 0.01. Bar graph showing the 10 most up-regulated and 10 most down-regulated predicted upstream regulators, when comparing WT and *CALR* DEL HOM cluster 3 (HSC) (blue) and cluster 11 (MK) (red), as measured by combining the *z* scores from WT and MK analyses.

Last, we made use of our scRNAseq data to compare gene expression between WT and *CALR* DEL HOM cells along the MK trajectory. We considered cells within 2 of the 13 clusters defined by our transcriptomic data (HSC and MK; fig. S1A) and 1 fine cluster (pMKP; arrow in Fig. 1A) (Fig. 6, A to C). As the pMKP fine cluster had fewer cells (24 in WT and 247 in *CALR* DEL HOM) than the larger HSC and MK clusters, we were only able to confidently call a small number of differentially expressed genes (DEGs) within this cluster. We performed Ingenuity Pathway Analysis (IPA) to determine which biological pathways and upstream regulators were most affected in the HSC and MK clusters; the small numbers of DEGs in pMKPs resulted in no statistically significant hits via IPA. The most affected canonical pathways fell into three broad groups: cell cycle (in blue), unfolded protein response (gold), and cholesterol biosynthesis (green) (Fig. 6, D and E). Full lists of canonical pathways, *P* values, and *z* scores are available in tables S1 (HSC) and S2 (MK). Genes contributing to these three pathways are highlighted in the same colors in Fig. 6, A to C; we note that pMKPs also show up-regulation of several UPR (unfolded protein response)–associated genes—such as *Hspa5*, *Pdia3*, and *Pdia6*—in addition to two known STAT targets (*Ifitm2* and *Socs2*).

While cell cycle and UPR have previously been described as up-regulated in human CD34^+^ cells with *CALR* mutation (*34*), the discovery of cholesterol biosynthesis was somewhat unexpected. However, this aligned with the predicted significant activation of the lipid and cholesterol biosynthetic transcriptional machinery controlled by the sterol regulatory element–binding proteins (SREBPs; SREBF1 and SREBF2) and the SREBF chaperone (SCAP) and their inhibitor insulin-induced gene 1 (INSIG1) (Fig. 6F). Moreover, as discussed further below, a role for cholesterol biosynthesis in a proliferative, platelet-biased blood disorder is biologically plausible. Upstream regulator analysis also pointed to activation of ERN1 (Ire1α) and Xbp1, two constituents of UPR, as well as STAT5 (table S3), which is consistent with previous demonstrations that mutant CALR acts via STAT signaling (*4*, *35*–*37*). We additionally observed other previously undescribed signaling processes to be predicted to be activated, including drivers of proliferation such as CSF2 [granulocyte-macrophage colony-stimulating factor (GM-CSF)] and hepatocyte growth factor (HGF), or repressed, like the known tumor suppressors TP53 and let-7.

## DISCUSSION

Single-cell transcriptomic approaches have allowed detailed examinations of differentiation landscapes in both normal and perturbed hematopoiesis without a requirement to initially define populations based on a set of cell surface markers. We therefore used single-cell transcriptomics to investigate our recently generated mutant CALR-driven mouse model of ET and found an expected increase in both HSCs and MK lineage cells. We also found an increase in a previously unknown group of cells, here termed pMKPs, linking HSCs with the MK lineage. In vitro, pMKPs displayed behaviors intermediate to those of HSCs and MkPs: Similarly to HSCs, they had some proliferative potential, but similarly to MkPs, they were almost exclusively restricted to the MK lineage. In transplantations, pMKPs and MkPs showed similar behavior: They both transiently produced platelets at a low level. We hypothesize that while pMKPs are more proliferative than MkPs in vitro, neither population is capable of sufficient proliferation to significantly contribute to platelet production in the transplant setting. While this manuscript was in preparation, another group described separating SLAM (Lin^−^ CD48^−^ CD150^+^) cells based on EPCR and CD34, finding that EPCR^−^ SLAM cells performed poorly in transplants and showed gene expression profiles (high *Gata1*, *Vwf*, and *Itga2b*) indicative of MK bias (*38*), results that are broadly consistent with our own.

Our characterization of pMKPs accords well with an increasing understanding that at least a portion of megakaryopoiesis occurs via an early branch point directly from HSCs. While the standard model of hematopoiesis shows megakaryocytes subsequent to MPP2, lineage tracing experiments have shown that some MkPs are generated in an MPP2-independent way (*19*). Furthermore, in vivo labeling of the most primitive HSCs showed that within 1 week of label induction in LT-HSCs, label can be seen in MK lineages but no other, indicating that the HSC-to-MK pathway can be noticeably faster than pathways producing other lineages (*22*). Our results suggest that pMKPs are likely to arise independently of the MPP2 stage, as suicidal depletion of the earliest HSPCs reduces pMKPs to a much greater extent than MPP2s. It is therefore tempting to speculate that our pMKP sort scheme may isolate intermediate cells on this shorter, faster bypass trajectory. A recent study of JAK2 *V617F*-driven MF in humans attributed increased megakaryopoiesis to the expansion of both traditional MkPs and a novel MkP-like population, suggesting that cells that may be analogous to our pMKPs are relevant in human disease (*30*).

We also investigated an outstanding question about at which stages mutant CALR acts to drive a platelet phenotype. Mutant CALR has been demonstrated to increase the number of immunophenotypic HSCs and MkPs (*6*), and we also saw an expansion in the number of pMKPs. When considering the behavior of cells individually, it is clear that mutant CALR acts from the stem cell compartment: CALR DEL HOM HSCs were more proliferative and faster to produce megakaryocytes than were their WT counterparts. Mutant CALR did not show a strong effect on the proliferation or MK bias of pMKPs at the level of a single cell but drove an increase in proliferation of MkPs and thus the number of megakaryocytes produced. We therefore concluded that mutant CALR drives platelet bias and proliferation at multiple stages of megakaryopoiesis, although this effect is strongest within HSCs.

Last, we used our single-cell transcriptomic data to ask which biological pathways were most differentially regulated in our *CALR* DEL HOM mice. Mutant CALR was associated with an up-regulation of the unfolded protein response, as would be expected for cells with impaired chaperone activity and as has been seen in human patient cells (*34*). In addition, mutant CALR cells showed an increase in cell cycle genes, again consistent with observations from human patient cells (*34*) and in agreement with our in vitro data, which showed that mutant CALR HSCs and MkPs were more proliferative. We also found up-regulation of cholesterol biosynthesis pathway genes in mutant CALR hematopoietic cells. While cholesterol biosynthesis is broadly increased across numerous cancers (*39*), including hematological cancers (*40*), CALR has also been directly linked to cholesterol biosynthesis. *CALR*^−/−^ mouse embryonic fibroblasts show impaired endoplasmic reticulum (ER) Ca^2+^ levels, leading to overactivation of SREBPs, which then up-regulate cholesterol and triacylglycerol biosynthesis genes (*41*). As mutant CALR lacks its Ca^2+^-binding domain, it is possible that *CALR* DEL HOM cells phenocopy knockout cells with respect to ER Ca^2+^ stores, thus leading to the observed overactive transcription of cholesterol biosynthesis genes. While megakaryocytes derived from human patient samples have been shown to have increased store-operated Ca^2+^ entry due to the perturbation of a complex between STIM1, ERp57, and CALR (*42*), none of our differentially activated pathways from IPA pointed to altered cytoplasmic Ca^2+^ signaling in the stem and progenitor populations tested. This may reflect differences between progenitor and mature cells. Mice with impaired cholesterol efflux have more proliferative HSCs (*43*) and an increase in MkP proliferation and an ET-like phenotype (*44*), suggesting that there may be a previously unknown link between the *CALR* DEL mutation, cholesterol metabolism, proliferation of MkPs, and thus the overproduction of platelets. While cholesterol biosynthesis was the most prominent novel target found in our transcriptomic analysis, it was by no means alone. IPA upstream regulator analysis predicted an up-regulation of interleukin-5 (IL-5), GM-CSF, and HGF—all with known roles in hematopoiesis—in addition to several unexpected results, such as TBX2, a transcription factor that has not been studied in hematopoiesis. Upstream regulators predicted to be decreased include the tumor suppressor TP53; let-7, a microRNA with a role in the self-renewal of fetal HSCs (*45*); and KDM5B (Jarid1b), a histone methylase required for HSC self-renewal (*46*).

Overall, our study has characterized a previously undescribed MK trajectory implicated in the progression of ET. We find that pMKPs are an intermediate stage within one pathway of megakaryopoiesis and hypothesize that they may be situated within the MPP2-independent MK shortcut. Last, our analysis confirmed that JAK-STAT signaling, unfolded protein response, and cell cycle are all increased by the presence of mutant CALR and found up-regulation of cholesterol biosynthesis, in addition to numerous other potential upstream regulators. Functional validation of these biological pathways and upstream regulators may represent promising avenues of future research to better understand mutant CALR-driven disease and in the development of therapeutic strategies.

## MATERIALS AND METHODS

### Experimental design

The objectives of the study were to generate transcriptomic data from our *CALR* mouse model of ET and to use these data to determine how the hematopoietic landscape is affected by the *CALR* DEL mutation. All mouse procedures were performed in strict accordance with the U.K. Home Office regulations for animal research under project license 70/8406.

### FACS sorting

Bone marrow cells were harvested from the femurs, tibia, and iliac crests of mice. Bones were crushed in a mortar and pestle in phosphate-buffered saline (PBS) and 2% fetal bovine serum (FBS) and 5 mM EDTA and then filtered through a 70-μm filter to obtain a suspension of bone marrow cells. The suspension was incubated with an equal volume of ammonium chloride solution (STEMCELL Technologies, Vancouver, Canada) for 10 min on ice to lyse erythrocytes, followed by centrifugation for 5 min at 350*g*. The cell pellet was resuspended in PBS and 2% FBS and 5 mM EDTA, filtered again through a 70-μm filter, and centrifuged again for 5 min at 350*g*. For cell sorting experiments, bone marrow mononuclear cell suspensions were immunomagnetically depleted of lineage (Lin)–positive cells (EasySep Mouse Hematopoietic Progenitor Cell Isolation Kit, catalog no. 19856, STEMCELL Technologies). For staining, cells were incubated with the indicated antibodies for 40 min on ice; see attached tables for catalog information and concentrations used (table S4). Flow cytometry was performed on BD LSRFortessa analyzers, and flow cytometric sorting was performed on BD Influx 4 and 5 cell sorters (BD Biosciences, San Jose, USA). Flow data were analyzed using FlowJo software (Tree Star, Ashland, USA).

### 10x data processing and quality control

For 10x Chromium (10x Genomics, Pleasanton, CA) experiments, Lin^−^ c-Kit^+^ (LK) and Lin^−^ Sca1^+^ cKit^+^ (LSK) cells were sort purified as described above and processed according to the manufacturer’s protocol. Sample demultiplexing, barcodes processing, and gene counting were performed using the count commands from the Cell Ranger v1.3 pipeline (https://support.10xgenomics.com/single-cell-gene-expression/software/overview/welcome). After Cell Ranger processing, each sample (LK and LSK for WT and *CALR* HOM DEL) was filtered for potential doublets by simulating synthetic doublets from pairs of scRNAseq profiles and assigning scores based on a *k* nearest-neighbor classifier on PCA-transformed data. The 1 and 4.5% of cells with the highest doublets scores from each LSK or LK sample were removed from further analysis, respectively. Cells with >10% of unique molecular identifier (UMI) counts mapping to mitochondrial genes, expressing fewer than 500 genes, or with a total number of UMI counts further than 3 SDs from the mean were excluded. After quality control, 11,098 WT (5139 LK and 5959 LSK) and 15,547 HOM (7815 LK and 7732 LSK) cells were retained for downstream analysis from our first repeat. For our second repeat, 3451 WT (2479 LK and 972 LSK) and 12,372 HOM (7824 LK and 4548 LSK) cells were retained for downstream analysis. These cells were then normalized to the same total count. All scRNAseq data were analyzed using the Scanpy Python Module (*47*).

### Clustering analysis and mapping

To assign cell type identities to WT and CALR samples, a previously published landscape of 45,000 WT LK and LSK hematopoietic progenitors (*24*) was used as a reference for cell type annotation. This reference was clustered using Louvain clustering, resulting in 13 clusters. LK + LSK samples were joined for each genotype (WT and *CALR* DEL HOM) and projected into the PCA space of this reference dataset. Nearest neighbors were calculated between the two datasets based on Euclidean distance in the top 50 PCA components. Cells were assigned to the same cluster to which the majority of their 15 nearest neighbors in the reference belonged.

### Force-directed graph visualization

A force-directed graph visualization of the 45,000 cell reference dataset was calculated by first constructing a *k* = 7 nearest-neighbor graph from the data, which was then used as input for the ForceAtlas2 algorithm as implemented in Gephi 0.9.1 (https://gephi.org). In the ForceAtlas2 algorithm, all cells are pushed away from each other, with the nearest-neighbor connections pulling them back together to segregate separate trajectories.

### PAGA graph visualization

A fine-resolution clustering of the reference dataset was calculated using the Louvain algorithm, resulting in 63 clusters. These were used as input for a PAGA analysis of the reference dataset using the Scanpy Python Module with default parameters. The results of the PAGA analysis were visualized by using the nodes and their edge weights as input into the ForceAtlas2 algorithm for calculating force-directed graphs as implemented in Gephi 0.9.1. For visualization, only connections with edge weights of >0.3 were shown.

### Marker gene expression on PAGA graph

To visualize gene expression of the PAGA graph, the mean normalized expression of all cells belonging to each node was calculated and displayed on a per-node basis.

### Differential abundance analysis

To calculate differential abundances, votes were given out from each WT LK and *CALR* LK cell to their *k*-nearest neighbors in the reference dataset, with *k* chosen such that the total number of votes given out by each sample was the same. For each cell in the reference dataset, the difference between the number of votes received from the WT and *CALR* HOM samples was calculated. This difference acts as a proxy for the differential abundance of WT and *CALR* HOM cells for the region of the LK landscape in which the reference cell is located. This differential abundance proxy could then be visualized either on the reference landscape itself or on the PAGA graph calculated using the reference landscape. In the latter case, each node of the PAGA graph was colored by the mean differential abundance of all cells belonging to that node.

### In vitro cell culture

After flow sorting, cells were cultured in StemSpan SFEM (serum-free expansion medium) (STEMCELL Technologies) supplemented with 10% FBS (STEMCELL Technologies), 1% penicillin/streptomycin (Sigma-Aldrich), 1% l-glutamine (Sigma-Aldrich), stem cell factor (SCF; 250 ng/ml), IL-3 (10 ng/ml), and IL-6 (10 ng/ml; STEMCELL Technologies), with or without thrombopoietin (100 ng/ml; STEMCELL Technologies), in round-bottom 96-well plates (Corning, Corning, USA). For pro-erythroid conditions, cells were cultured as above but with the following cytokines: SCF (250 ng/ml), THPO (thrombopoietin) (50 ng/ml), EPO (erythropoietin) (5 U/ml), IL-3 (20 ng/ml), and Flt3L (50 ng/ml). For pro-myeloid conditions, cells were cultured as above but with the following cytokines: SCF (250 ng/ml), THPO (50 ng/ml), granulocyte colony-stimulating factor (50 ng/ml), IL-3 (20 ng/ml), Flt3L (50 ng/ml), and GM-CSF (50 ng/ml).

### Classification of single-cell clones

At 1, 2, 3, 4, and, in some cases, 7 days after flow sorting, single cell–derived clones were visually inspected. Wells with surviving cells were classified into one of four categories: (i) exactly one enlarged cell, presumed to be a megakaryocyte; (ii) multiple enlarged cells; (iii) mixed expansion, with both small and enlarged cells; and (iv) expansion with only small cells. In some cases, the experimenter was blinded to the identity of the cell population initially sorted into the well he/she was inspecting and the genotype of the mouse.

### Fluorescence microscopy

For immunofluorescence, cells were allowed to adhere to the surface of poly-l-lysine–coated slides for 30 min at 37°C (Poly-Prep Slides, Sigma-Aldrich). Cells were then fixed with 4% paraformaldehyde (Sigma-Aldrich) in PBS overnight at 4°C, permeabilized with 0.25% Triton X-100 (Sigma-Aldrich) in PBS for 10 min at room temperature, and blocked with 1% bovine serum albumin (Sigma-Aldrich) for 1 hour at room temperature. Cells were stained with CD41 Alexa Fluor 488 (BioLegend, catalog no. 133908) overnight and mounted with 4′,6-diamidino-2-phenylindole (DAPI) (VECTASHIELD Mounting Medium with DAPI, Vector Laboratories Inc., Burlingame, USA; catalog no. H-1500). Pictures were acquired on LSM-710 and LSM-780 confocal microscopes (Zeiss) and analyzed using ZEN software (Zeiss). For quantification of immunofluorescence, cells were cultured on CD44-coated glass-bottom plates for immobilization (*48*), followed by fixation and staining as above. Pictures were acquired on a Leica DMI4000 microscope (Leica), and CD41 intensity and cell size were quantified using Fiji software.

### Bone marrow transplantation

FACS-sorted cells from VWF-GFP^+^ donors were injected into the tail veins of W41/W41 (CD45.1) recipient that had been sublethally irradiated with 1 × 400 centigrays with 250,000 spleen cells as helpers. Peripheral blood was analyzed 1, 2, 3, 4, and 16 weeks after transplant for all cohorts.

### Differential gene expression

Differential expression analysis was performed between WT (LK + LSK) and CALR DEL HOM (LK + LSK) clusters using the Wilcoxon rank sum test on all genes that passed initial quality control (typically approximately 15,000). A Benjamini-Hochberg correction was applied to correct for multiple testing. Genes with an adjusted *P* value of <0.05 and a fold change of >1.5 between genotypes were marked as differentially expressed. The original normalized counts were used in all cases.

### Ingenuity Pathway Analysis

DEGs were studied using IPA (Qiagen). We imputed the whole transcriptome in IPA and then filtered for analysis only statistically significant (adjusted *P* < 0.01) items with a log_2_FC > 0.3785 or log_2_FC < −0.3785. “Pathways” and “upstream regulator” networks showing relationships and interactions experimentally confirmed between DEGs and others that functionally interact with them were generated and ranked in terms of significance of participating genes (*P* < 0.05) and activation status (*z* score).

### Statistical analysis

Data were analyzed, and graphs were generated in Microsoft Excel (Microsoft) and GraphPad PRISM (GraphPad, La Jolla, USA). Data are presented as means ± SD. Unless otherwise stated, statistical tests were unpaired Student’s *t* tests. *P* values are as follows: *P < 0.05, **P < 0.01, ***P < 0.001, and ****P < 0.0001.

## Supplementary Material

http://advances.sciencemag.org/cgi/content/full/6/48/eabd3139/DC1

Table S1

Table S2

Table S3

Table S4

Adobe PDF - abd3139_SM.pdf

The stem/progenitor landscape is reshaped in a mouse model of esential thrombocythemia and causes exces megakaryocyte production

## SUPPLEMENTARY MATERIALS

Supplementary material for this article is available at http://advances.sciencemag.org/cgi/content/full/6/48/eabd3139/DC1

## Appendix

View/request a protocol for this paper from *Bio-protocol*.

## References

[1] Grinfeld J., Nangalia J., Green A. R., Molecular determinants of pathogenesis and clinical phenotype in myeloproliferative neoplasms. Haematologica 102, 7–17 (2017). 27909216 10.3324/haematol.2014.113845 PMC5210228

[2] Nangalia J., Massie C. E., Baxter E. J., Nice F. L., Gundem G., Wedge D. C., Avezov E., Li J., Kollmann K., Kent D. G., Aziz A., Godfrey A. L., Hinton J., Martincorena I., Van Loo P., Jones A. V., Guglielmelli P., Tarpey P., Harding H. P., Fitzpatrick J. D., Goudie C. T., Ortmann C. A., Loughran S. J., Raine K., Jones D. R., Butler A. P., Teague J. W., O’Meara S., McLaren S., Bianchi M., Silber Y., Dimitropoulou D., Bloxham D., Mudie L., Maddison M., Robinson B., Keohane C., Maclean C., Hill K., Orchard K., Tauro S., Du M.-Q., Greaves M., Bowen D., Huntly B. J. P., Harrison C. N., Cross N. C. P., Ron D., Vannucchi A. M., Papaemmanuil E., Campbell P. J., Green A. R., Somatic *CALR* mutations in myeloproliferative neoplasms with nonmutated *JAK2*. N. Engl. J. Med. 369, 2391–2405 (2013). 24325359 10.1056/NEJMoa1312542 PMC3966280

[3] Klampfl T., Gisslinger H., Harutyunyan A. S., Nivarthi H., Rumi E., Milosevic J. D., Them N. C. C., Berg T., Gisslinger B., Pietra D., Chen D., Vladimer G. I., Bagienski K., Milanesi C., Casetti I. C., Sant’Antonio E., Ferretti V., Elena C., Schischlik F., Cleary C., Six M., Schalling M., Schönegger A., Bock C., Malcovati L., Pascutto C., Superti-Furga G., Cazzola M., Kralovics R., Somatic mutations of calreticulin in myeloproliferative neoplasms. N. Engl. J. Med. 369, 2379–2390 (2013). 24325356 10.1056/NEJMoa1311347

[4] Elf S., Abdelfattah N. S., Chen E., Perales-Patón J., Rosen E. A., Ko A., Peisker F., Florescu N., Giannini S., Wolach O., Morgan E. A., Tothova Z., Losman J. A., Schneider R. K., Al-Shahrour F., Mullally A., Mutant calreticulin requires both its mutant C-terminus and the thrombopoietin receptor for oncogenic transformation. Cancer Discov. 6, 368–381 (2016). 26951227 10.1158/2159-8290.CD-15-1434 PMC4851866

[5] Nivarthi H., Chen D., Cleary C., Kubesova B., Jäger R., Bogner E., Marty C., Pecquet C., Vainchenker W., Constantinescu S. N., Kralovics R., Thrombopoietin receptor is required for the oncogenic function of CALR mutants. Leukemia 30, 1759–1763 (2016). 26883579 10.1038/leu.2016.32 PMC4980558

[6] Li J., Prins D., Park H. J., Grinfeld J., Gonzalez-Arias C., Loughran S., Dovey O. M., Klampfl T., Bennett C., Hamilton T. L., Pask D. C., Sneade R., Williams M., Aungier J., Ghevaert C., Vassiliou G. S., Kent D. G., Green A. R., Mutant calreticulin knockin mice develop thrombocytosis and myelofibrosis without a stem cell self-renewal advantage. Blood 131, 649–661 (2018). 29282219 10.1182/blood-2017-09-806356

[7] Eaves C., Hematopoietic stem cells: Concepts, definitions, and the new reality. Blood 125, 2605–2614 (2015). 25762175 10.1182/blood-2014-12-570200 PMC4440889

[8] M. Kondo, I. L. Weissman, K. Akashi, Identification of clonogenic common lymphoid progenitors in mouse bone marrow. 91, 661–672 (1997). 10.1016/s0092-8674(00)80453-5 9393859

[9] K. Akashi, D. Traver, T. Miyamoto, A clonogenic common myeloid progenitor that gives rise to all myeloid lineages. 404, 193–197 (2000). 10.1038/35004599 10724173

[10] Nestorowa S., Hamey F. K., Pijuan Sala B., Diamanti E., Shepherd M., Laurenti E., Wilson N. K., Kent D. G., Göttgens B., A single-cell resolution map of mouse hematopoietic stem and progenitor cell differentiation. Blood 128, e20–e31 (2016). 27365425 10.1182/blood-2016-05-716480 PMC5305050

[11] Paul F., Arkin Y., Giladi A., Jaitin D. A., Kenigsberg E., Keren-Shaul H., Winter D., Lara-Astiaso D., Gury M., Weiner A., David E., Cohen N., Lauridsen F. K. B., Haas S., Schlitzer A., Mildner A., Ginhoux F., Jung S., Trumpp A., Porse B. T., Tanay A., Amit I., Transcriptional heterogeneity and lineage commitment in myeloid progenitors. Cell 163, 1663–1677 (2015). 26627738 10.1016/j.cell.2015.11.013

[12] Tusi B. K., Wolock S. L., Weinreb C., Hwang Y., Hidalgo D., Zilionis R., Waisman A., Huh J. R., Klein A. M., Socolovsky M., Population snapshots predict early haematopoietic and erythroid hierarchies. Nat. Publ. Gr. 555, 54–60 (2018). 10.1038/nature25741 PMC5899604 29466336

[13] Müller-Sieburg C. E., Cho R. H., Thoman M., Adkins B., Sieburg H. B., Muller-Sieburg C. E., Cho R. H., Thoman M., Adkins B., Sieburg H. B., Müller-Sieburg C. E., Cho R. H., Thoman M., Adkins B., Sieburg H. B., Deterministic regulation of hematopoietic stem cell self-renewal and differentiation. Blood 100, 1302–1309 (2002). 12149211

[14] Muller-Sieburg C. E., Cho R. H., Karlsson L., Huang J. F., Sieburg H. B., Myeloid-biased hematopoietic stem cells have extensive self-renewal capacity but generate diminished lymphoid progeny with impaired IL-7 responsiveness. Blood 103, 4111–4118 (2004). 14976059 10.1182/blood-2003-10-3448

[15] Dykstra B., Kent D., Bowie M., McCaffrey L., Hamilton M., Lyons K., Lee S. J., Brinkman R., Eaves C., Long-term propagation of distinct hematopoietic differentiation programs in vivo. Cell Stem Cell 1, 218–229 (2007). 18371352 10.1016/j.stem.2007.05.015

[16] Adolfsson J., Månsson R., Buza-Vidas N., Hultquist A., Liuba K., Jensen C. T., Bryder D., Yang L., Borge O. J., Thoren L. A. M., Anderson K., Sitnicka E., Sasaki Y., Sigvardsson M., Jacobsen S. E. W., Identification of Flt3+ lympho-myeloid stem cells lacking erythro-megakaryocytic potential: A revised road map for adult blood lineage commitment. Cell 121, 295–306 (2005). 15851035 10.1016/j.cell.2005.02.013

[17] Månsson R., Hultquist A., Luc S., Yang L., Anderson K., Kharazi S., Al-Hashmi S., Liuba K., Thorén L., Adolfsson J., Buza-Vidas N., Qian H., Soneji S., Enver T., Sigvardsson M., Jacobsen S. E. W., Molecular evidence for hierarchical transcriptional lineage priming in fetal and adult stem cells and multipotent progenitors. Immunity 26, 407–419 (2007). 17433729 10.1016/j.immuni.2007.02.013

[18] Sanjuan-Pla A., Macaulay I. C., Jensen C. T., Woll P. S., Luis T. C., Mead A., Moore S., Carella C., Matsuoka S., Jones T. B., Chowdhury O., Stenson L., Lutteropp M., Green J. C. A., Facchini R., Boukarabila H., Grover A., Gambardella A., Thongjuea S., Carrelha J., Tarrant P., Atkinson D., Clark S. A., Nerlov C., Jacobsen S. E. W., Platelet-biased stem cells reside at the apex of the haematopoietic stem-cell hierarchy. Nature 502, 232–236 (2013). 23934107 10.1038/nature12495

[19] Rodriguez-Fraticelli A. E., Wolock S. L., Weinreb C. S., Panero R., Patel S. H., Jankovic M., Sun J., Calogero R. A., Klein A. M., Camargo F. D., Clonal analysis of lineage fate in native haematopoiesis. Nature 553, 212–216 (2018). 29323290 10.1038/nature25168 PMC5884107

[20] Chapple R. H., Tseng Y., Hu T., Kitano A., Takeichi M., Hoegenauer K. A., Nakada D., Lineage tracing of murine adult hematopoietic stem cells reveals active contribution to steady-state hematopoiesis. Blood Adv. 2, 1220–1228 (2018). 29848758 10.1182/bloodadvances.2018016295 PMC5998934

[21] Sawai C. M., Babovic S., Upadhaya S., Knapp D. J. H. F., Lavin Y., Lau C. M., Goloborodko A., Feng J., Fujisaki J., Ding L., Mirny L. A., Merad M., Eaves C. J., Reizis B., Hematopoietic stem cells are the major source of multilineage hematopoiesis in adult animals. Immunity 45, 597–609 (2016). 27590115 10.1016/j.immuni.2016.08.007 PMC5054720

[22] Upadhaya S., Sawai C. M., Papalexi E., Rashidfarrokhi A., Jang G., Chattopadhyay P., Chattopadhyay P., Satija R., Reizis B., Kinetics of adult hematopoietic stem cell differentiation in vivo. J. Exp. Med. 215, 2815–2832 (2018). 30291161 10.1084/jem.20180136 PMC6219744

[23] Säwen P., Eldeeb M., Erlandsson E., Kristiansen T. A., Laterza C., Kokaia Z., Karlsson G., Yuan J., Soneji S., Mandal P. K., Rossi D. J., Bryder D., Murine HSCs contribute actively to native hematopoiesis but with reduced differentiation capacity upon aging. eLife 7, e41258 (2018). 30561324 10.7554/eLife.41258 PMC6298771

[24] Dahlin J. S., Hamey F. K., Pijuan-Sala B., Shepherd M., Lau W. W. Y., Nestorowa S., Weinreb C., Wolock S., Hannah R., Diamanti E., Kent D. G., Göttgens B., Wilson N. K., A single-cell hematopoietic landscape resolves 8 lineage trajectories and defects in Kit mutant mice. Blood 131, e1–e11 (2018). 29588278 10.1182/blood-2017-12-821413 PMC5969381

[25] Wolf F. A., Hamey F. K., Plass M., Solana J., Dahlin J. S., Göttgens B., Rajewsky N., Simon L., Theis F. J., PAGA: Graph abstraction reconciles clustering with trajectory inference through a topology preserving map of single cells. Genome Biol. 20, 59 (2019). 30890159 10.1186/s13059-019-1663-x PMC6425583

[26] A. B. Balazs, A. J. Fabian, C. T. Esmon, R. C. Mulligan, Endothelial protein C receptor ( CD201 ) explicitly identifies hematopoietic stem cells in murine bone marrow. 107, 2317–2321 (2006). 10.1182/blood-2005-06-2249 PMC1895725 16304059

[27] Pronk C. J. H., Bryder D., Immunophenotypic identification of early myeloerythroid development. Methods Mol. Biol. 1678, 301–319 (2018). 29071684 10.1007/978-1-4939-7346-0_13

[28] Shin J. Y., Hu W., Naramura M., Park C. Y., High c-Kit expression identifies hematopoietic stem cells with impaired self-renewal and megakaryocytic bias. J. Exp. Med. 211, 217–231 (2014). 24446491 10.1084/jem.20131128 PMC3920569

[29] Roch A., Trachsel V., Lutolf M. P., Brief report: Single-cell analysis reveals cell division-independent emergence of megakaryocytes from phenotypic hematopoietic stem cells. Stem Cells 33, 3152–3157 (2015). 26184464 10.1002/stem.2106

[30] Psaila B., Wang G., Rodriguez-Meira A., Li R., Heuston E. F., Murphy L., Yee D., Hitchcock I. S., Sousos N., O’Sullivan J., Anderson S., Senis Y. A., Weinberg O. K., Calicchio M. L., Iskander D., Royston D., Milojkovic D., Roberts I., Bodine D. M., Thongjuea S., Mead A. J., Single-cell analyses reveal megakaryocyte-biased hematopoiesis in myelofibrosis and identify mutant clone-specific targets. Mol. Cell 78, 477–492.e8 (2020). 32386542 10.1016/j.molcel.2020.04.008 PMC7217381

[31] Pronk C. J. H., Rossi D. J., Månsson R., Attema J. L., Norddahl G. L., Chan C. K. F., Sigvardsson M., Weissman I. L., Bryder D., Elucidation of the phenotypic, functional, and molecular topography of a myeloerythroid progenitor cell hierarchy. Cell Stem Cell 1, 428–442 (2007). 18371379 10.1016/j.stem.2007.07.005

[32] Schoedel K. B., Morcos M. N. F., Zerjatke T., Roeder I., Grinenko T., Voehringer D., Göthert J. R., Waskow C., Roers A., Gerbaulet A., The bulk of the hematopoietic stem cell population is dispensable for murine steady-state and stress hematopoiesis. Blood 128, 2285–2296 (2016). 27357698 10.1182/blood-2016-03-706010

[33] Pietras E. M., Reynaud D., Kang Y. A., Carlin D., Calero-Nieto F. J., Leavitt A. D., Stuart J. A., Göttgens B., Passegué E., Functionally distinct subsets of lineage-biased multipotent progenitors control blood production in normal and regenerative conditions. Cell Stem Cell 17, 35–46 (2015). 26095048 10.1016/j.stem.2015.05.003 PMC4542150

[34] Nam A. S., Kim K.-T., Chaligne R., Izzo F., Ang C., Taylor J., Myers R. M., Abu-Zeinah G., Brand R., Omans N. D., Alonso A., Sheridan C., Mariani M., Dai X., Harrington E., Pastore A., Cubillos-Ruiz J. R., Tam W., Hoffman R., Rabadan R., Scandura J. M., Abdel-Wahab O., Smibert P., Landau D. A., Somatic mutations and cell identity linked by Genotyping of Transcriptomes. Nature 571, 355–360 (2019). 31270458 10.1038/s41586-019-1367-0 PMC6782071

[35] Marty C., Pecquet C., Nivarthi H., El-Khoury M., Chachoua I., Tulliez M., Villeval J. L., Raslova H., Kralovics R., Constantinescu S. N., Plo I., Vainchenker W., Calreticulin mutants in mice induce an MPL-dependent thrombocytosis with frequent progression to myelofibrosis. Blood 127, 1317–1324 (2016). 26608331 10.1182/blood-2015-11-679571

[36] Chachoua I., Pecquet C., El-Khoury M., Nivarthi H., Albu R. I., Marty C., Gryshkova V., Defour J. P., Vertenoeil G., Ngo A., Koay A., Raslova H., Courtoy P. J., Choong M. L., Plo I., Vainchenker W., Kralovics R., Constantinescu S. N., Thrombopoietin receptor activation by myeloproliferative neoplasm associated calreticulin mutants. Blood 127, 1325–1335 (2016). 26668133 10.1182/blood-2015-11-681932

[37] Araki M., Yang Y., Masubuchi N., Hironaka Y., Takei H., Morishita S., Mizukami Y., Kan S., Shirane S., Edahiro Y., Sunami Y., Ohsaka A., Komatsu N., Activation of the thrombopoietin receptor by mutant calreticulin in CALR-mutant myeloproliferative neoplasms. Blood 127, 1307–1316 (2016). 26817954 10.1182/blood-2015-09-671172

[38] Rabe J. L., Hernandez G., Chavez J. S., Mills T. S., Nerlov C., Pietras E. M., CD34 and EPCR coordinately enrich functional murine hematopoietic stem cells under normal and inflammatory conditions. Exp. Hematol. 81, 1–15.e6 (2020). 31863798 10.1016/j.exphem.2019.12.003 PMC6938677

[39] Kuzu O. F., Noory M. A., Robertson G. P., The role of cholesterol in cancer. Cancer Res. 76, 2063–2070 (2016). 27197250 10.1158/0008-5472.CAN-15-2613 PMC5813477

[40] Oguro H., The roles of cholesterol and its metabolites in normal and malignant hematopoiesis. Front. Endocrinol. 10, 204 (2019). 10.3389/fendo.2019.00204 PMC6454151 31001203

[41] Wang W. A., Liu W. X., Durnaoglu S., Lee S. K., Lian J., Lehner R., Ahnn J., Agellon L. B., Michalak M., Loss of calreticulin uncovers a critical role for calcium in regulating cellular lipid homeostasis. Sci. Rep. 7, 5941 (2017). 28725049 10.1038/s41598-017-05734-x PMC5517566

[42] Di Buduo C. A., Abbonante V., Marty C., Moccia F., Rumi E., Pietra D., Soprano P., Lim D., Cattaneo D., Iurlo A., Gianelli U., Barosi G., Rosti V., Plo I., Cazzola M., Balduini A., Defective interaction of mutant calreticulin and SOCE in megakaryocytes from patients with myeloproliferative neoplasms. Blood 135, 133–144 (2019). 10.1182/blood.2019001103 PMC6952826 31697806

[43] Yvan-Charvet L., Pagler T., Gautier E. L., Avagyan S., Siry R. L., Han S., Welch C. L., Wang N., Randolph G. J., Snoeck H. W., Tall A. R., ATP-binding cassette transporters and HDL suppress hematopoietic stem cell proliferation. Science 328, 1689–1693 (2010). 20488992 10.1126/science.1189731 PMC3032591

[44] Murphy A. J., Bijl N., Yvan-Charvet L., Welch C. B., Bhagwat N., Reheman A., Wang Y., Shaw J. A., Levine R. L., Ni H., Tall A. R., Wang N., Cholesterol efflux in megakaryocyte progenitors suppresses platelet production and thrombocytosis. Nat. Med. 19, 586–594 (2013). 23584088 10.1038/nm.3150 PMC3683965

[45] Copley M. R., Babovic S., Benz C., Knapp D. J. H. F., Beer P. A., Kent D. G., Wohrer S., Treloar D. Q., Day C., Rowe K., Mader H., Kuchenbauer F., Humphries R. K., Eaves C. J., The Lin28b-let-7-Hmga2 axis determines the higher self-renewal potential of fetal haematopoietic stem cells. Nat. Cell Biol. 15, 916–925 (2013). 23811688 10.1038/ncb2783

[46] Stewart M. H., Albert M., Sroczynska P., Cruickshank V. A., Guo Y., Rossi D. J., Helin K., Enver T., The histone demethylase Jarid1b is required for hematopoietic stem cell self-renewal in mice. Blood 125, 2075–2078 (2015). 25655602 10.1182/blood-2014-08-596734 PMC4467872

[47] Wolf F. A., Angerer P., Theis F. J., SCANPY: Large-scale single-cell gene expression data analysis. Genome Biol. 19, 15 (2018). 29409532 10.1186/s13059-017-1382-0 PMC5802054

[48] Loeffler D., Wang W., Hopf A., Hilsenbeck O., Bourgine P. E., Rudolf F., Martin I., Schroeder T., Mouse and human HSPC immobilization in liquid culture by CD43- or CD44-antibody coating. Blood 131, 1425–1429 (2018). 29453290 10.1182/blood-2017-07-794131

