# Supplementary material for: The stem/progenitor landscape is reshaped in a mouse model of essential thrombocythemia and causes excess megakaryocyte production
Source: Sci Adv. 2020 Nov 25;6(48):eabd3139. doi: 10.1126/sciadv.abd3139 (PMC7688335; doi:10.1126/sciadv.abd3139)
Supplement: http://advances.sciencemag.org/cgi/content/full/6/48/eabd3139/DC1 [file supp_6_48_eabd3139__index.html]

Science Advances | Science AdvancesAAASSearchScience AdvancesMenu

## Supplementary Materials

# The stem/progenitor landscape is reshaped in a mouse model of essential thrombocythemia and causes excess megakaryocyte production

Daniel Prins, Hyun Jung Park, Sam Watcham, Juan Li, Michele Vacca, Hugo P. Bastos, Alexander Gerbaulet, Antonio Vidal-Puig, Berthold Göttgens, Anthony R. Green

Download Supplement

**This PDF file includes:**

- Figs. S1 to S6

**Other Supplementary Material for this manuscript includes the following:**

- Table S1
- Table S2
- Table S3
- Table S4

**Files in this Data Supplement:**

- Adobe PDF - abd3139\_SM.pdf
- abd3139\_Table\_S1.xlsx
- abd3139\_Table\_S2.xlsx
- abd3139\_Table\_S3.xlsx
- abd3139\_Table\_S4.xlsx
