# Supplementary material for: The stem/progenitor landscape is reshaped in a mouse model of essential thrombocythemia and causes excess megakaryocyte production
Source: Sci Adv. 2020 Nov 25;6(48):eabd3139. doi: 10.1126/sciadv.abd3139 (PMC7688335; doi:10.1126/sciadv.abd3139)
Supplement: http://advances.sciencemag.org/cgi/content/full/6/48/eabd3139/DC1 [file supp_6_48_eabd3139__1.pdf]

Science Advances
AAAS

advances.sciencemag.org/cgi/content/full/6/48/eabd3139/DC1

Supplementary Materials for

The stem/progenitor landscape is reshaped in a mouse model of essential thrombocythemia and causes excess megakaryocyte production

Daniel Prins, Hyun Jung Park, Sam Watcham, Juan Li, Michele Vacca, Hugo P. Bastos, Alexander Gerbaulet, Antonio Vidal-Puig, Berthold Göttgens, Anthony R. Green*

*Corresponding author. Email: arg1000@cam.ac.uk

Published 25 November 2020, *Sci. Adv.* **6**, eabd3139 (2020)
DOI: 10.1126/sciadv.abd3139

The PDF file includes:

Figs. S1 to S6

Other Supplementary Material for this manuscript includes the following:

(available at advances.sciencemag.org/cgi/content/full/6/48/eabd3139/DC1)

Tables S1 to S4

Supplemental Figure 1

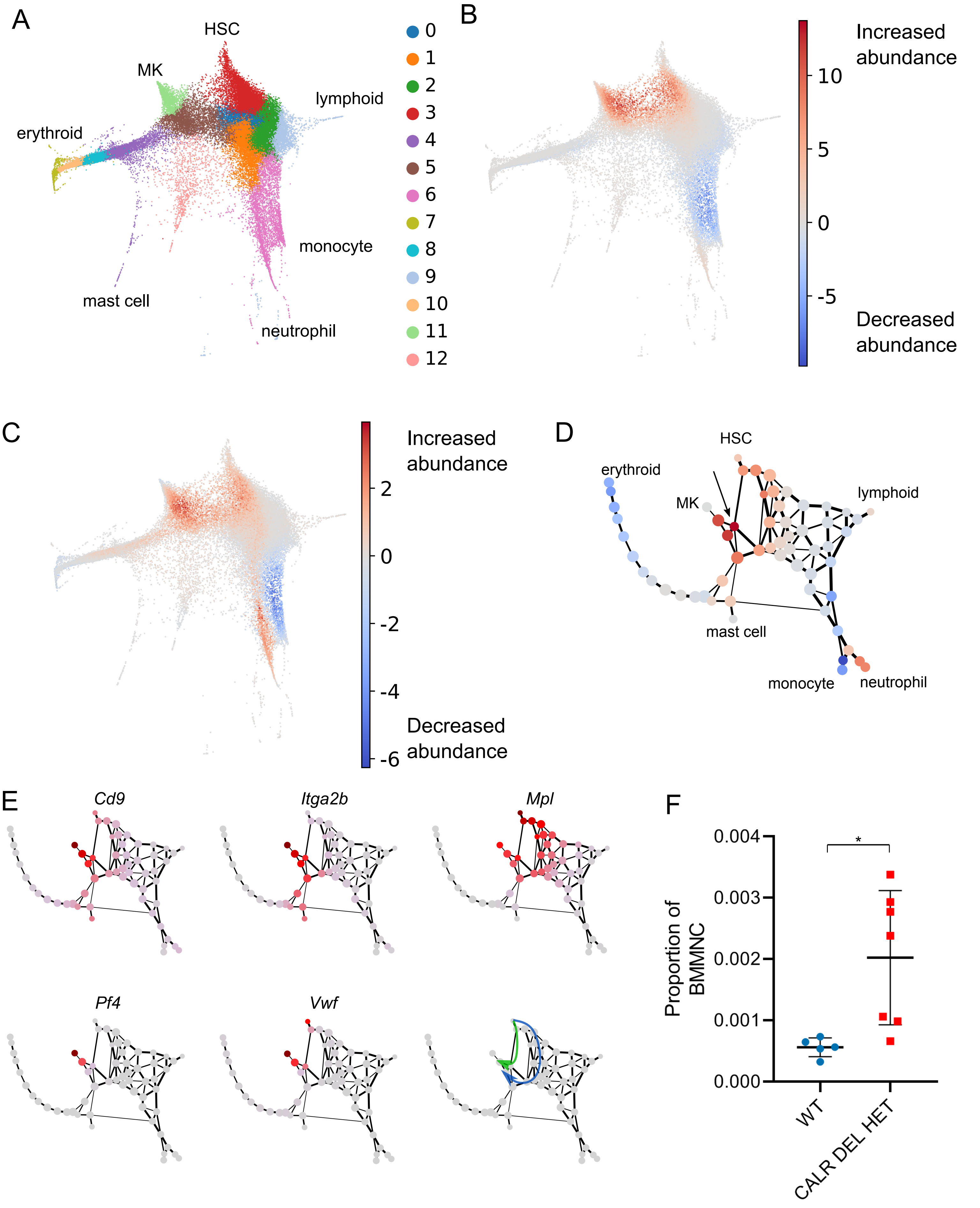

Supplemental Figure 1. Additional bioinformatics analysis.

A. Force-directed graph from Dahlin et al. (*24*) with LK and LSK cells classified into 13 different clusters based on scRNAseq data. **B, C.** scRNAseq data from LK and LSK cells from two independent repeats of WT and CALR DEL HOM mice, plotted on the force-directed graph from (A) and colored for relative enrichment (red) or depletion (blue) in CALR DEL HOM stem and progenitor cells. **D.** Partition-based graph abstraction (PAGA) as in Fig. 1A for the second repeat of WT and CALR DEL HOM mice, with the most highly enriched node marked with an arrow. **E.** Expression of the megakaryocytic markers *Cd9*, *Itga2b*, *Mpl*, *Pf4,* and *Vwf* from scRNAseq data, with two curved arrows representing proposed megakaryocytic trajectories. pMKPs are an intermediate in the green trajectory. **F.** Quantification of bone marrow frequency of pMKPs in WT and CALR DEL HET mice. The frequency of pMKPs within live bone marrow mononuclear cells (BMMNCs) is significantly increased in CALR DEL HET mice (WT, n=5, 0.00056 ± 0.00015; HOM, n=7, 0.0020 ± 0.0012; p=0.012).

Supplemental Figure 2

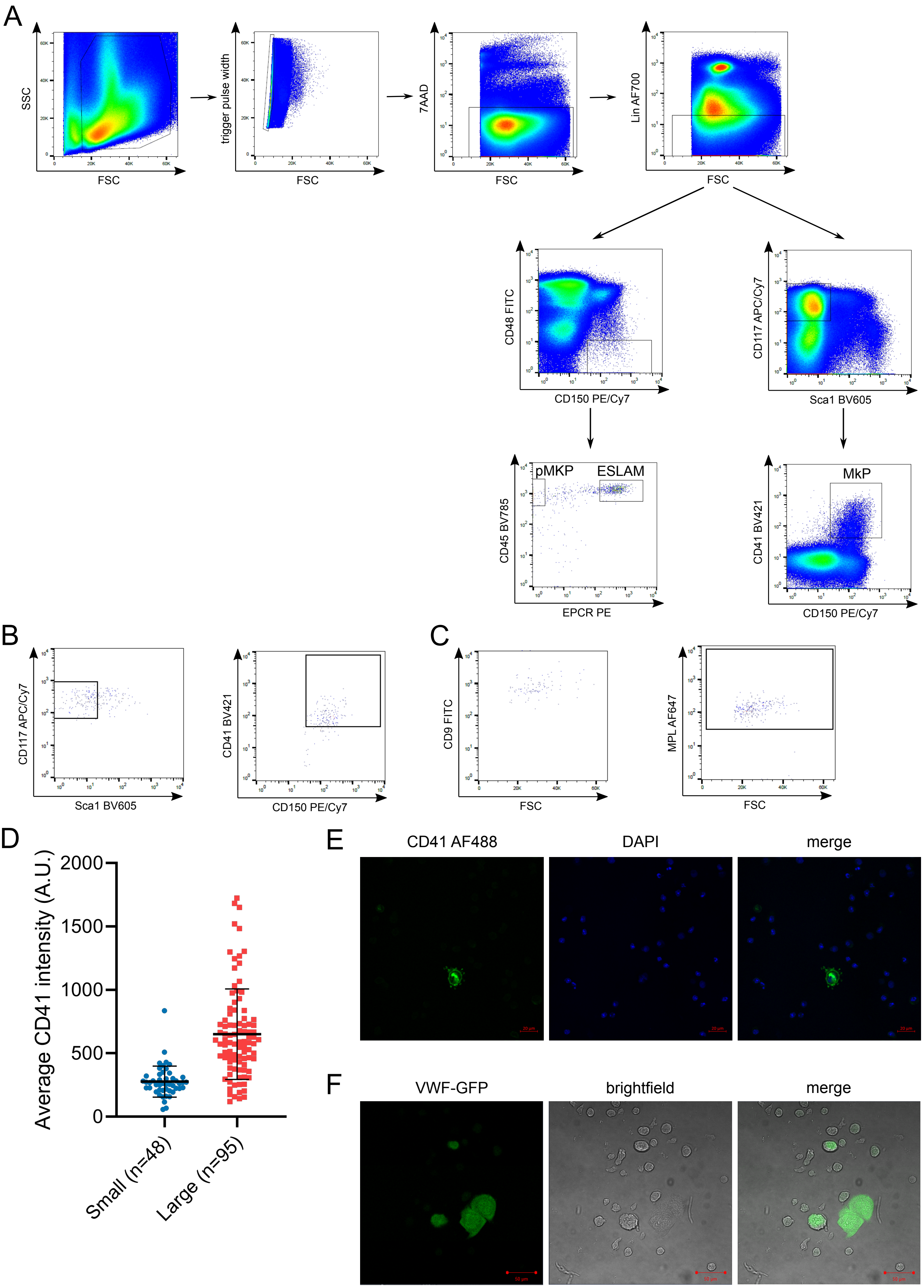

Supplemental Figure 2. Representative gating strategies for ESLAMs, pMKPs, and MkPs and immunofluorescence of cultured cells.

A. Representative gating strategies for FACS sorting ESLAMs, pMKPs (both on left), and MkPs (on right). **B.** pMKPs co-stained with MkP markers. pMKPs are typically $cKit^+$, with variable levels of Sca1 and medium to high levels of CD41. **C.** pMKPs co-stained with CD9 and MPL are uniformly $CD9^+$ and MPL^+. **D.** Quantification of CD41 average intensity from day 4 cells derived from ESLAMs, pMKPs, and MkPs, then classified as small or large. Large cells have significantly higher average CD41 intensity than do small cells (small: n=48, 277 ± 122 AU; large: n=95, 651 ± 356 AU; $p=7x10^{-16}$). **E**. Immunofluorescence staining of cells arising from ESLAMs in a mixed colony shows a noticeably large cell that is $CD41^+$ with membrane extensions resembling proplatelets. **F**. pMKPs from $VWF\text{-}GFP^+$ mice give rise to cells in a mixed colony that gain VWF-GFP fluorescence with increasing size.

Supplemental Figure 3

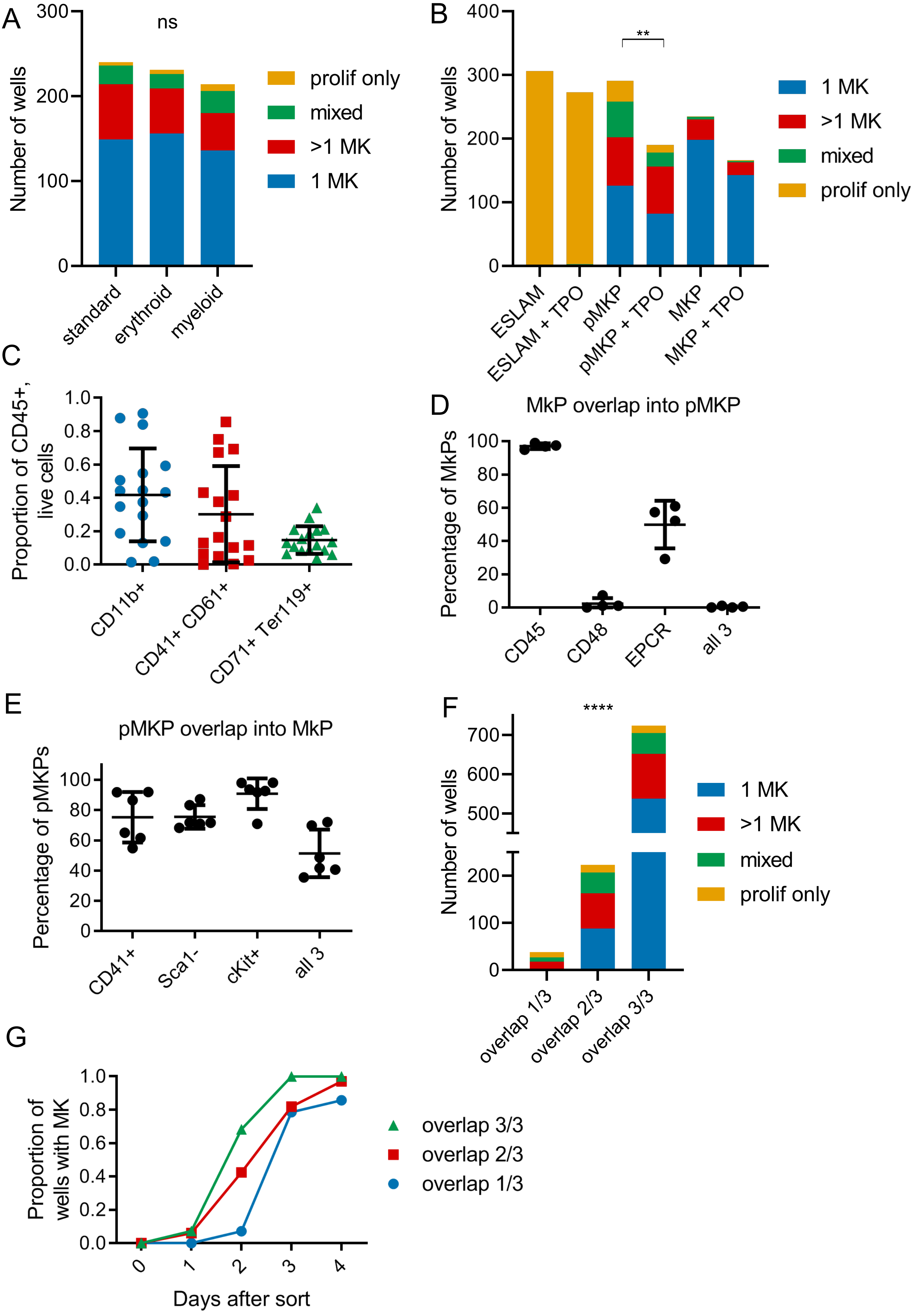

Supplemental Figure 3. Further analysis of pMKPs.

A. pMKPs maintain their MK bias even when cultured in pro-erythroid or pro-myeloid conditions. Standard n=240, erythroid n=231, myeloid n=214. Chi-square test, p=0.28. **B**. Effects of thrombopoietin treatment on ESLAMs, pMKPs, and MkPs. Thrombopoietin has an effect on pMKPs, increasing the proportion of wells that produce multiple MK. Chi-square test for pMKP vs pMKP + TPO, p=0.004. ESLAM n=306, ESLAM + TPO n=273, pMKP n=291, pMKP + TPO n=190, MkP n=235, MkP + TPO n=166. **C**. Culture conditions used to support pMKP survival allow multilineage differentiation of ESLAMs after 10 days in culture, n=17 per group. **D**. Overlap of immunophenotypic MkPs into the pMKP gate, based on index sort data. Most MkPs are $CD48^+$ (2% $CD48^-$) and are therefore not immunophenotypic pMKPs; n=4 separate experiments. **E**. Overlap of immunophenotypic pMKPs into the MkP gate, based on index sort data. pMKPs are 75% $CD41^+$, 75% $Sca1^-$, and 91% $cKit^+$; on average, 51% of pMKPs also fall within the MkP gate; n=6 separate experiments. **F**. pMKPs with the lowest overlap score are the most proliferative and least MK-biased, while those that are also immunophenotypically MkPs are the most MK-biased and least proliferative. 1/3 overlap score n=38, 2/3 overlap score n=223, 3/3 overlap score n=724. Chi-square test, p<0.0001. **G**. pMKPs with the lowest overlap score are the slowest to show evidence of megakaryopoiesis.

Supplemental Figure 4

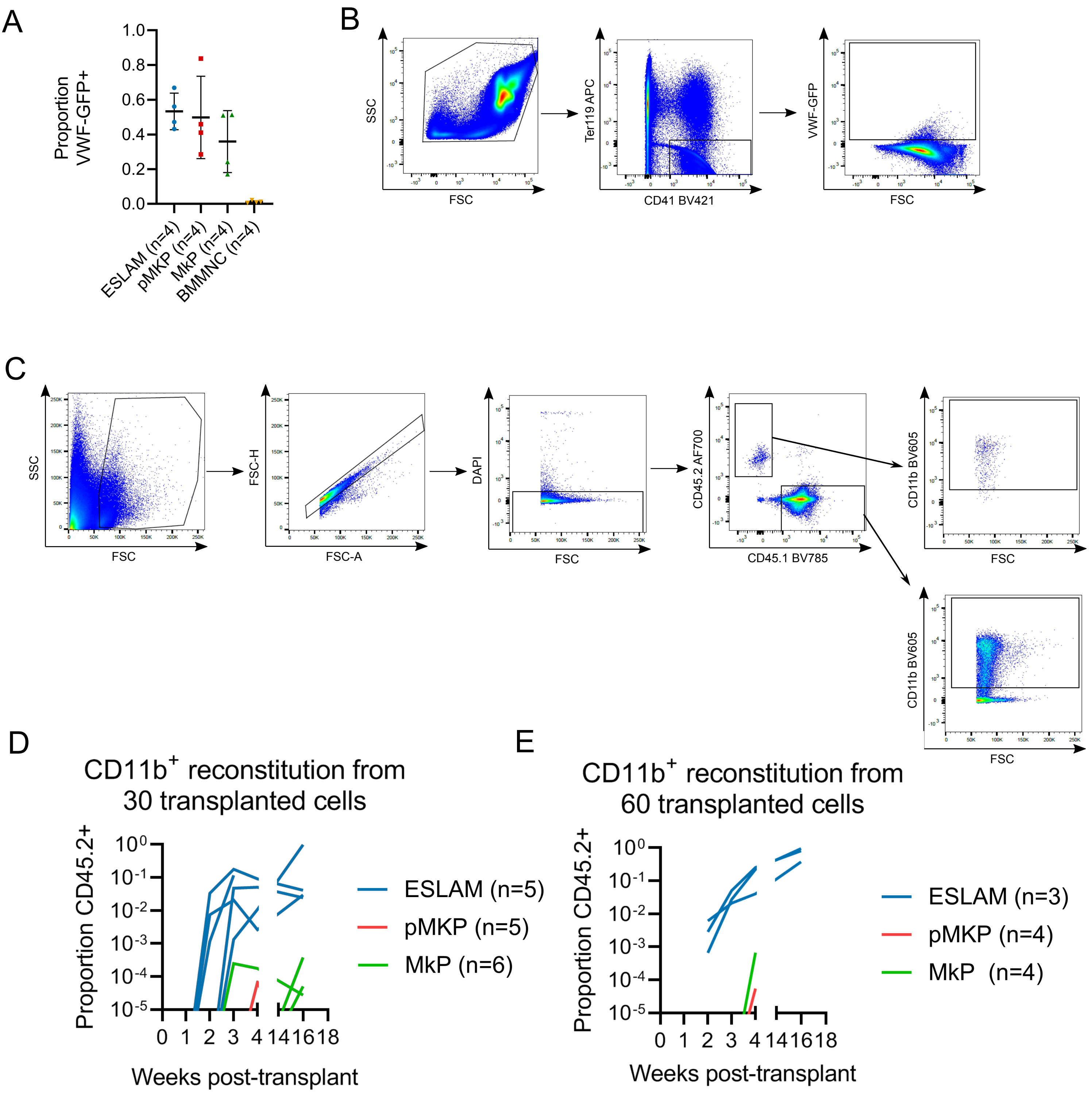

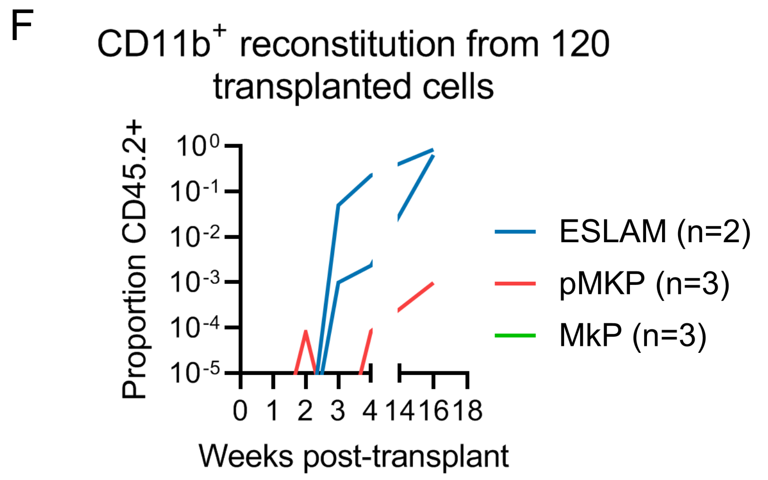

G

	ESLAM	pMKP	MkP
30 cells	5/5	1/5	1/6
60 cells	3/3	1/4	1/4
120 cells	2/2	1/3	0/3

Supplemental Figure 4. Representative flow plots for analysis of blood chimerism and analysis of myeloid blood chimerism.

A. Analysis of VWF-GFP positivity in ESLAMs, pMKPs, MkPs, and BMMNCs from mice used as donors for transplants. **B**. Representative flow plots for analysis of VWF-GFP plt chimerism. **C**. Representative flow plots for analysis of $CD45.2^+$ $CD11b^+$ chimerism. **D**. $CD11b^+$ (myeloid) reconstitution from 30 donor cells. **E**. $CD11b^+$ (myeloid) reconstitution from 30 donor cells. **F**. $CD11b^+$ (myeloid) reconstitution from 30 donor cells. **G**. Table summarizing numbers of mice with successful $CD11b^+$ production from ESLAMs, pMKPs, and MkPs. Here, transplanted cells were defined to have produced myeloid cells if $CD45.2^+$ $CD11b^+$ cells were observed at a level of at least 1 in 10^4 at one or more time points within the first four weeks post-transplantation.

Supplemental Figure 5

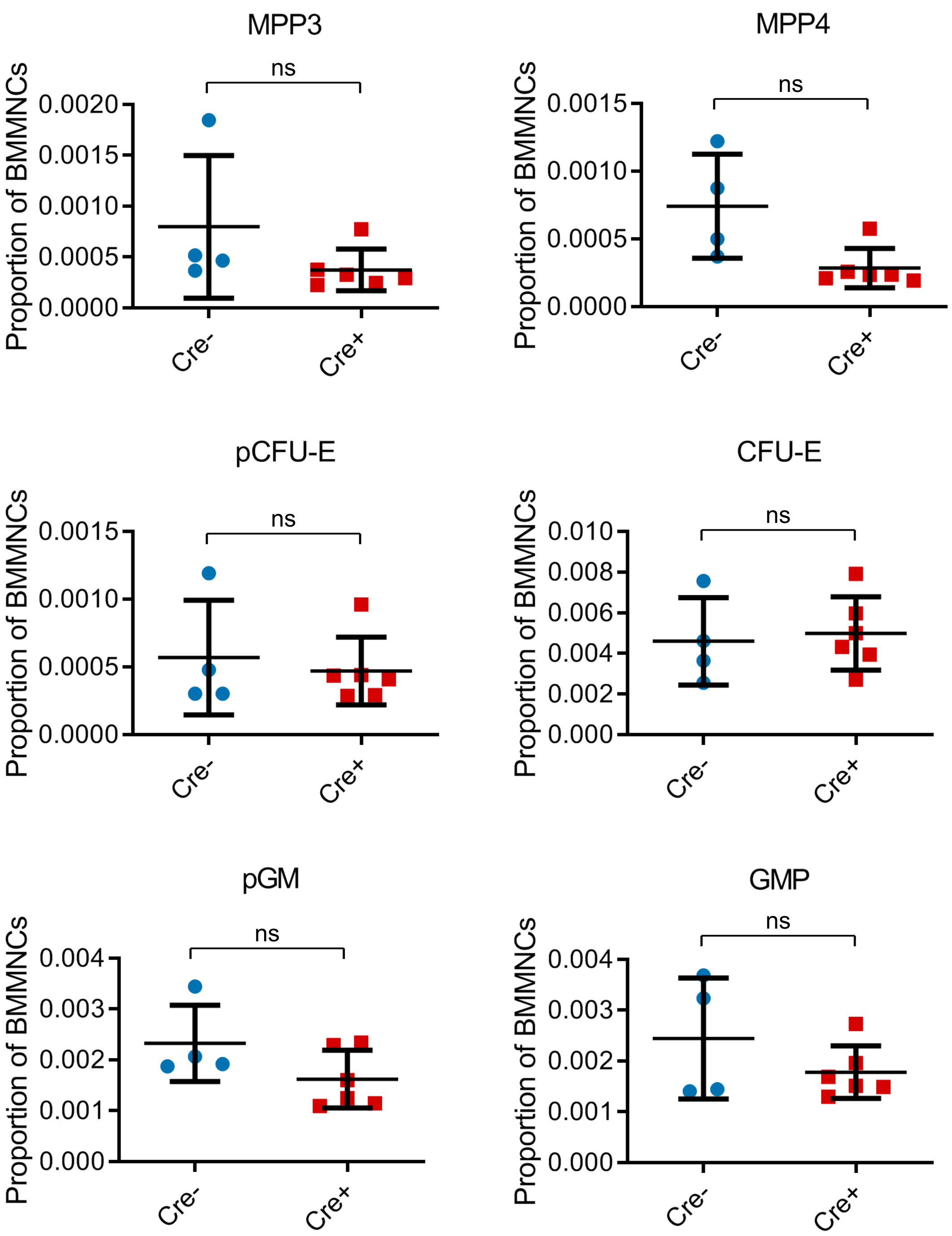

Supplemental Figure 5. Analysis of additional progenitor populations from DTA mice.

Frequencies of additional progenitor populations with and without stem cell depletion. MPP3 (Cre^-: 80 ± 70/10^5 BMMNC, Cre^+: 37 ± 20/10^5 BMMNC, p=0.32), MPP4 (Cre^-: 74 ± 38/10^5 BMMNC, Cre^+: 28 ± 14/10^5 BMMNC, p=0.094), pCFU-E (Cre^-: 57 ± 42/10^5 BMMNC, Cre^+: 47 ± 25/10^5 BMMNC, p=0.69), CFU-E (Cre^-: 460 ± 215/10^5 BMMNC, Cre^+: 497 ± 180/10^5 BMMNC, p=0.78), pGM (Cre^-: 232 ± 75/10^5 BMMNC, Cre^+: 162 ± 57/10^5 BMMNC, p=0.17), and GMP (Cre^-: 244 ± 119/10^5 BMMNC, Cre^+: 178 ± 52/10^5 BMMNC, p=0.35) did not show statistically significant differences with and without stem cell depletion. Cre^- n=4, Cre^+ n=6.

Supplemental Figure 6

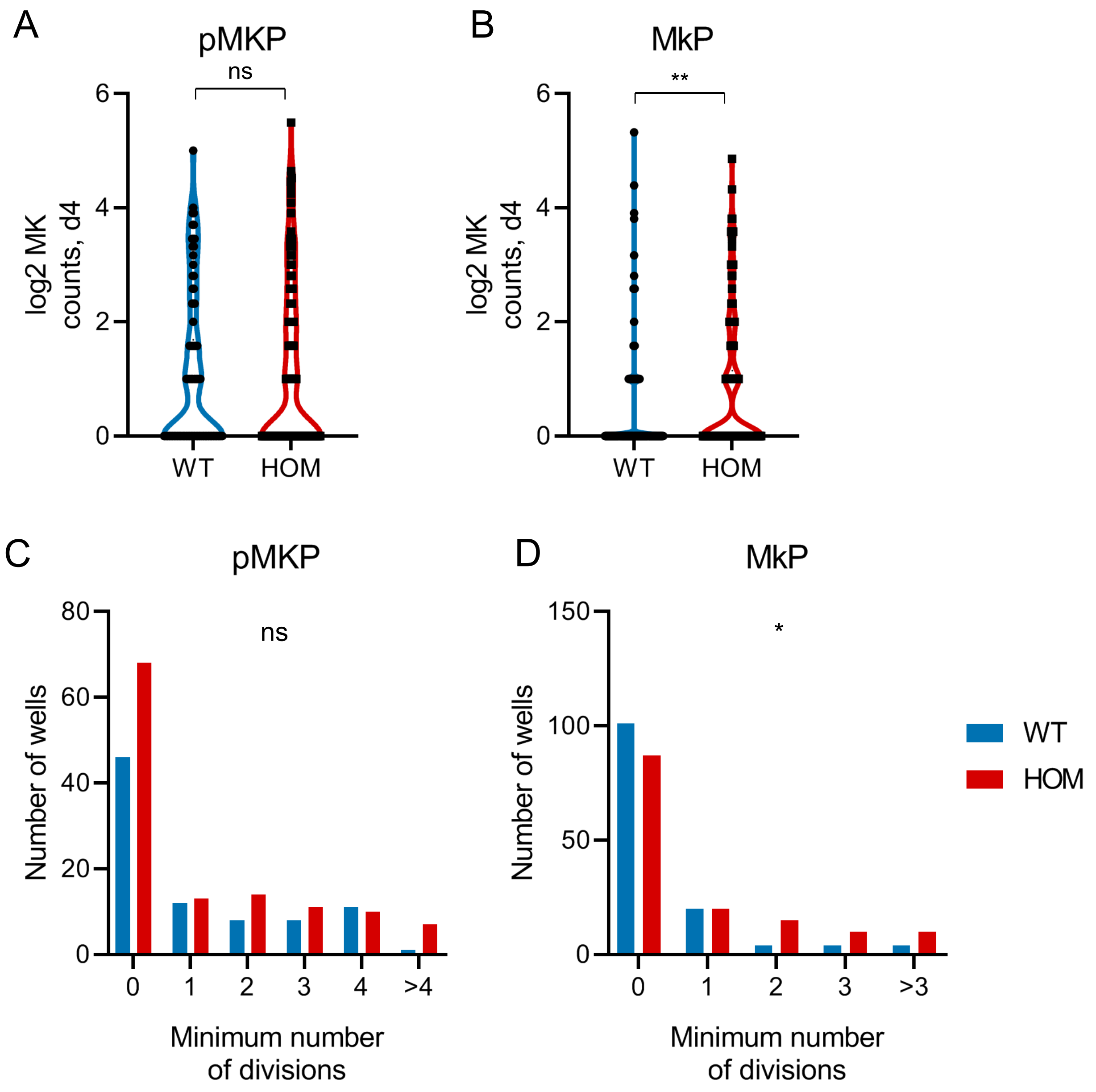

Supplemental Figure 6. Additional analysis of WT AND CALR DEL HOM ESLAMs, pMKPs, and MkPs.

A. Violin plots of log_2 transformed cell counts of megakaryocytes from WT and CALR DEL HOM pMKPs after 4 days of culture. Median values: WT=0, HOM=0; Mann-Whitney U = 5274; $n_1 = 86$, $n_2 = 123$, $p = 0.97$. **B**. Violin plots of log_2 transformed cell counts of megakaryocytes from WT and CALR DEL HOM MkPs after 4 days of culture. Median values: WT=0, HOM=0; Mann-Whitney U = 7979; $n_1 = 134$, $n_2 = 142$, $p = 0.0050$. **C**. Histogram of the minimum number of cell divisions for WT and CALR DEL HOM pMKPs after 4 days of culture. Chi-square test, $p = 0.4752$. **D**. Histogram of the minimum number of cell divisions for WT and CALR DEL HOM MkPs after 4 days of culture. Chi-square test, $p = 0.0154$.
